# Supplementary material for: Sharing pain: Using pain domain transfer for video recognition of low grade orthopedic pain in horses
Source: PLoS One. 2022 Mar 4;17(3):e0263854. doi: 10.1371/journal.pone.0263854 (PMC8896717; doi:10.1371/journal.pone.0263854)
Supplement: S1 File — (ZIP) [file pone.0263854.s001.zip › Supporting Information/Sharing_Pain_APPENDIX.pdf]

Appendix for

Sharing Pain: Using Pain Domain Transfer for Video Recognition of Low Grade Orthopedic Pain in Horses

Sofia Broomé [1*], Katrina Ask [2], Maheen Rashid-Engström [4,5], Pia Haubro Andersen [3], Hedvig Kjellström [1,6]

1 Division of Robotics, Perception and Learning, KTH Royal Institute of Technology, Stockholm, Sweden
2 Department of Anatomy, Physiology and Biochemistry, Swedish University of Agricultural Sciences, Uppsala, Sweden
3 Department of Clinical Sciences, Swedish University of Agricultural Sciences, Uppsala, Sweden
4 Department of Computer Science, University of California, Davis, USA
5 Univrses, Stockholm, Sweden
6 Silo AI, Stockholm, Sweden

* sbroome@kth.se

M, E	A	B	H	I	J	K	N	Global
**I3D** [3], Kinetics								
PF †, 63	54.96	45.05	53.42	56.52	55.69	41.66	46.59	52.70
PF, 25	51.0 ±0.9	46.7 ±0.63	45.5 ±7.33	52.1 ±1.8	56.0 ±4.23	41.5 ±0.43	46.2 ±2.4	51.7 ±1.4
PF, 63	49.9 ±2.8	48.0 ±2.1	47.2 ±2.6	52.2 ±1.8	59.0 ±1.0	40.7 ±0.8	46.1 ±2.2	52.6 ±0.4
PF, 115	49.8 ±1.8	48.1 ±1.5	45.1 ±1.8	54.0 ±0.8	58.9 ±1.3	40.2 ±0.8	47.0 ±1.1	52.6 ±0.05
PF, 200	50.2 ±2.2	46.9 ±0.9	50.2 ±1.8	51.1 ±1.4	58.5 ±0.6	41.3 ±0.2	47.1 ±1.4	52.4 ±0.5
**C-LSTM-2**, scratch								
PF †, 115	61.55	56.34	55.55	51.51	64.76	45.11	57.84	58.17
PF, 115	59.4 ±4.6	56.7 ±2.7	60.5 ±5.7	52.1 ±1.8	53.2 ±14.0	49.6 ±3.9	54.2 ±4.2	56.3 ±2.8

Table 1. Repetitions (three repetitions whenever a mean and standard deviation are presented) of the results in Table 4 of the main article, and supplementary runs for I3D for a varying number of epochs. M for model, E for epochs, A, B, H, I, J, K, N for different horse subjects, † for the instance used in the main article.

A Supplementary material for the human expert study

In Figs 1-2, we show RGB, optical flow and Grad-CAM [1] saliency maps for each of the classification decisions on clip 1-25 taken by C-LSTM-2-PF †. The clips are the same as the ones listed in Table 7 of the main article.

B Supplementary experiments

B.1 Domain transfer results for additional model instances

Table 1 contains results for repeated runs of training C-LSTM-2 on the entire PF dataset for 115 epochs. Importantly, it contains further results on I3D trained on the entire PF dataset for a varied number of epochs. This it to make sure that we did not miss out on better performing behavior of I3D in our comparison.

The reason for the slight discrepancy between the global F1-scores and the subject-wise F1-scores is due to the varying proportions of pain and non-pain clips between the subjects. In the global setting, the classes are approximately balanced due to the resampling procedure (Section C.1.1). Since the F1-score is the harmonic mean of precision and recall, a non-linear function, it does not simply behave as a mean when it is computed for different parts of a training set.

B.2 Cross-validation within one domain on smaller frames

Table 2 shows cross-validated intra-domain results for C-LSTM-2 when the input resolution is 128x128 pixels. This is the same input resolution as in [2], but the training was conducted along with the training modifications listed in Section C.1 (except for the frame resolution). The observation that treating weak labels as dense labels works for acute pain (PF) but not for orthopedic pain (EOP(j)), where the expressions are sparse over time, still holds for input data of this size.

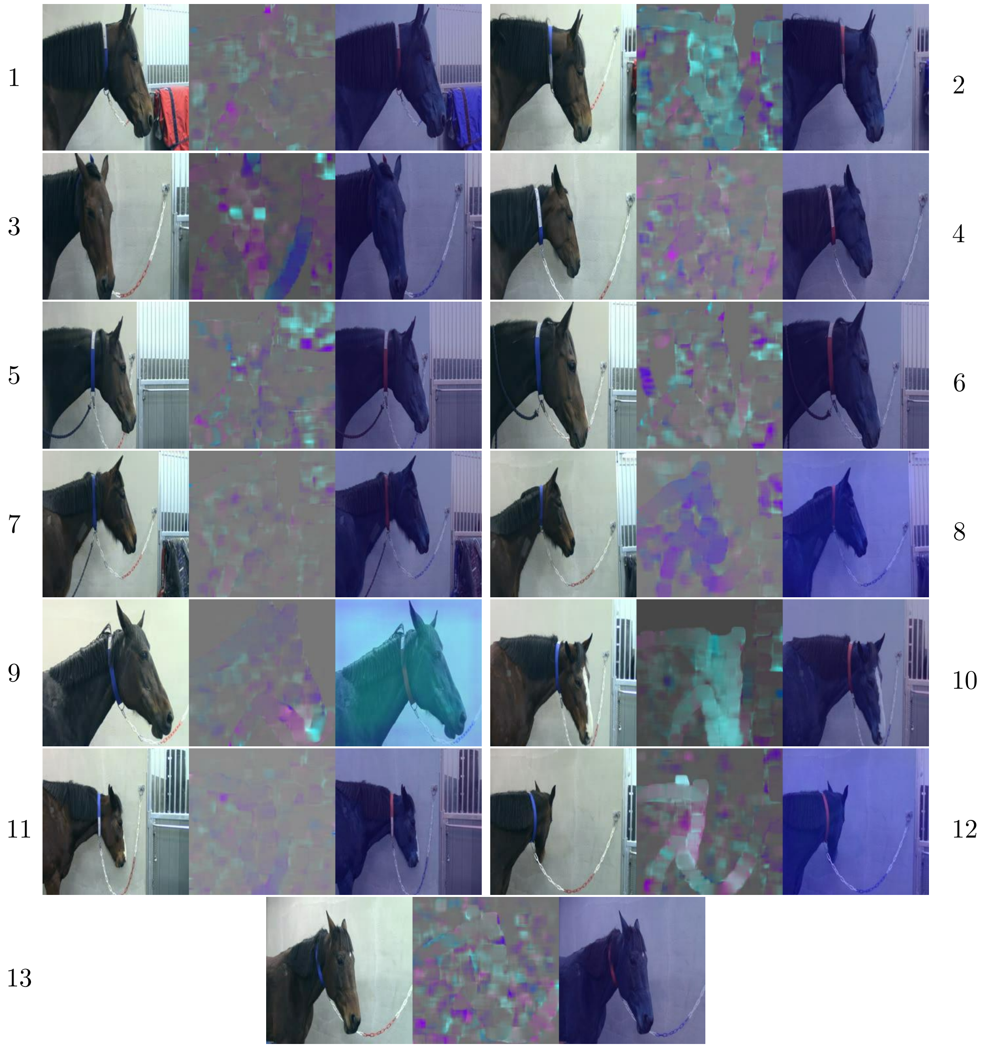

Fig 1. Every pain-clip from the human expert baseline (clips 1-13). **The figures can be viewed as animations in the Supporting Information, or on click in Adobe reader. Here, only the first frame of each sequence is shown.** RGB (*left*), optical flow (*middle*), and Grad-CAM [1] saliency maps (*right*) of the C-LSTM-2-PF † predictions on clips 1-13 (Table 7 in the main article).

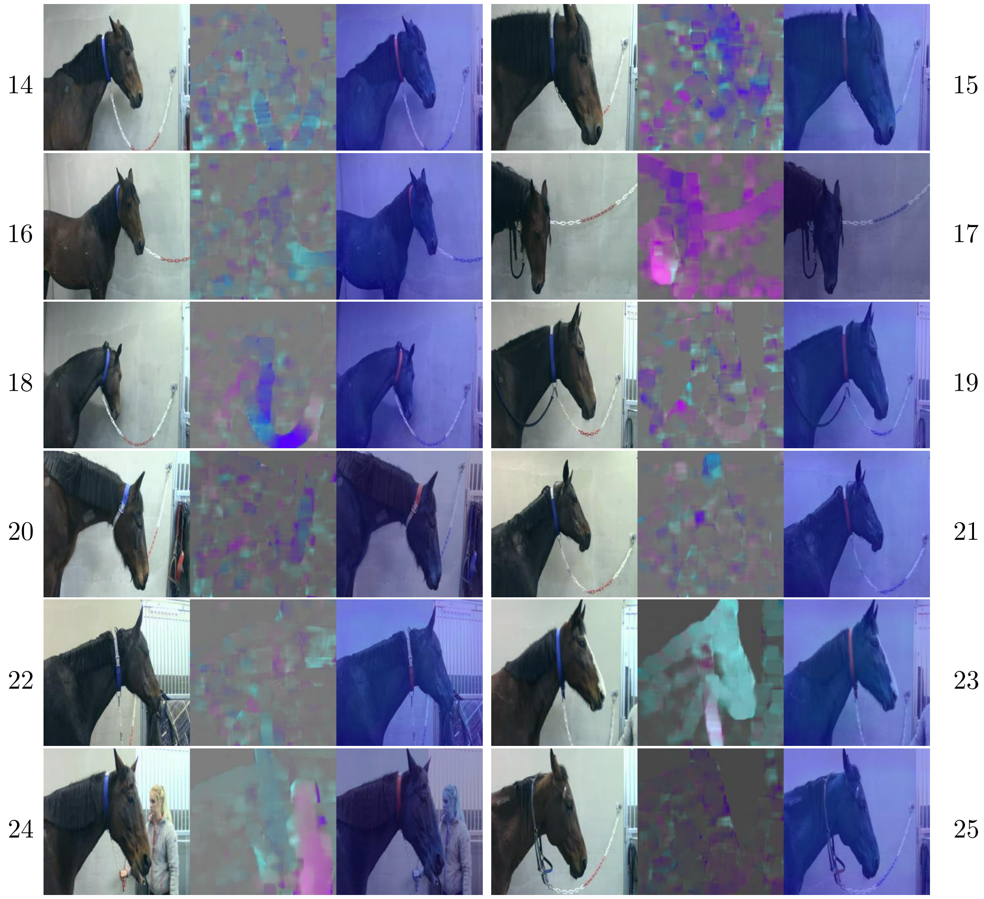

Fig 2. Every non-pain-clip from the human expert baseline (clips 14-25). **The figures can be viewed as animations in the Supporting Information, or on click in Adobe reader. Here, only the first frame of each sequence is shown.** RGB (*left*), optical flow (*middle*), and Grad-CAM [1] saliency maps (*right*) of the C-LSTM-2-PF † predictions on clips 14-25 (Table 7 in the main article).

Dataset	Horse folds	F1-score	Accuracy
PF	6	80.7 ±4.4	81.2 ±3.9
EOP(j)	7	48.3 ±3.6	49.4 ±3.2
PF + EOP(j)*	13	58.5 ±4.5	60.3 ±4.1
(*PF		70.8 ±4.1	73.0 ±4.1)
(*EOP(j)		48.0 ±4.8	49.3 ±4.2)

Table 2. Results (%) on 128x128 frames from training on clip-level for the respective datasets using the C-LSTM-2. The total result is the average of five repetitions of a full cross-validation and the average of the per-subject-across-runs standard deviations.

C Training details

C.1 Modified training of the C-LSTM-2 [2]

We follow the supervised training protocol of [2] but make the following modifications.

- We run on 224x224x3 frames, instead of 128x128x3. This forces us to use a batch size of 2 instead of 8 on a GPU with 11GB memory (the sequence length is kept at 10). However, Table 2 shows cross-validation results for C-LSTM-2 on 128x128 resolution on the two datasets (batch size 8).
- Each clip is horizontally flipped with 0.5 probability, as data augmentation.
- Clip-level binary cross-entropy is used during optimization, instead of on frame-level as in [2]. The test results in [2] were presented as majority votes across clips. Here, we use clip-level classifications during both training, validation and testing.
- The RGB data is standardized according to the pixel mean and standard deviation of the dataset. The optical flow data in the two-stream model is linearly scaled to the 0-1 range from the 0-255 jpg range, to have similar magnitude as the RGB data.
- Since there is class imbalance, we resample the minor class (i.e., we extract sequences more frequently across a video, details in Section C.1.1). This is done during validation and testing as well, for easier interpretation of the results.
- We train for maximum 200 epochs, with 50 epochs early stopping based on the validation set, and use the model state at the epoch with best performance on the validation set to run on the held-out test subject. In [2], the maximum number of epochs was 100, with 15 epochs early stopping.

C.1.1 Algorithm for clip resampling

Clip resampling of the minor class (typically the pain class) was introduced to have class balance. Frames from the videos are extracted at 2fps. The clips consist of windows of frames with a certain window length w_L, extracted with some window stride w_S, across a video. Before resampling, $w_L = 10$ and $w_S = 10$ (back-to-back window extraction), and the start index for extraction $t_{start} = 0$. When resampling, in order to obtain a number of clips from the same video which are maximally different from the previously extracted clips, we want to sample starting from the index $t_{start} = \frac{w_L}{2}$, which in this case equals five.

For each training/validation/test split, we sample as many clips as required to have an equal number of pain and non-pain clips:
$n_{resample} = \text{abs}(n_{minor\ class} - n_{major\ class})$. We resample $n_{resample}/M$ clips per video, where M is the total number of videos of a dataset. The code used for the resampling can be found in the public repository.

References

1. Selvaraju RR, Cogswell M, Das A, Vedantam R, Parikh D, Batra D. Grad-CAM: Visual Explanations from Deep Networks via Gradient-Based Localization. Proceedings of the IEEE International Conference on Computer Vision. 2017;2017-Octob:618–626. doi:10.1109/ICCV.2017.74.

2. Broomé S, Gleerup KB, Andersen PH, Kjellström H. Dynamics Are Important for the Recognition of Equine Pain in Video. In: The IEEE Conference on Computer Vision and Pattern Recognition (CVPR); 2019.

3. Carreira J, Zisserman A. Quo Vadis, Action Recognition? A New Model and the Kinetics Dataset. In: CVPR; 2017.
